# The Impact of Interprofessional Monitoring and Education on the Usage of Systemic Glucocorticoids in Acute Exacerbations of Chronic Obstructive Pulmonary Disease: A Retrospective, Medication Use Review

**DOI:** 10.7759/cureus.6224

**Published:** 2019-11-24

**Authors:** Nicholas L Biondi, Michael M Samiratedu, Emily Highsmith, Adam Rosenblum, Kerri McGrady, Savannah Knepper, Riley Bowers

**Affiliations:** 1 Internal Medicine, Cape Fear Valley Health System, Fayetteville, USA; 2 Pharmacy, Cape Fear Valley Health System, Fayetteville, USA; 3 Internal Medicine, Campbell University School of Osteopathic Medicine, Fayetteville, USA; 4 Pharmacy, Cape Fear Valley Health, Fayetteville, USA; 5 Pharmacy, Campbell University College of Pharmacy & Health Sciences, Buies Creek, USA

**Keywords:** copd exacerbation, quality improvement, learner-centric research, interprofessional education, medical education, graduate medical education, chronic obstructive pulmonary disease (copd), pulmonology, corticosteroids

## Abstract

Background

Systemic corticosteroid therapy for chronic obstructive pulmonary disease (COPD) exacerbations is routine in clinical practice, however, dosing is variable. The Global Initiative for Chronic Obstructive Lung Disease (GOLD) panel recommends a short course of systemic corticosteroids for acute COPD exacerbation treatment. Despite these recommendations, institutions continue to use higher doses and longer durations of systemic corticosteroid therapies.

Methods

This single-center, retrospective, cohort study evaluated systemic corticosteroid use in inpatient treatment of COPD exacerbations. Data were collected on patients with a diagnosis of COPD exacerbation from October 2017 to February 2018 in both the control and education groups. An interprofessional, learner-centric, quality improvement, educational seminar was performed. Providers were given accompanying pocket reference material for improved adherence to GOLD guidelines for the management of acute COPD exacerbations.

Results

Of the 137 charts reviewed in the control group, 130 of 137 patients (94.9%) received systemic corticosteroid doses exceeding GOLD guideline recommendations. These patients received an average daily dose of 147.5 mg of prednisone equivalents. These patients also experienced more adverse drug reactions as compared to their post-intervention counterparts. The 105 charts examined post-educational intervention revealed 47 of 105 patients (44.8%) received GOLD guideline-directed doses of systemic corticosteroids. This was an improvement from 2.9% (4 of 137) in the control group (p-value < 0.001). The average daily dose decreased to 58 mg daily (p-value < 0.001), and the number of doses over the recommended 40 mg of prednisone equivalents (54 of 105) was a 43.5% reduction (p-value < 0.001). Length of stay also decreased in the education group from 6.1 +/- 4.1 to 4.7 +/- 2.8 days (p-value 0.009). The 30-day readmission rate, however, was not statistically different between the two groups, 31.4% pre- and 21.0% post-educational intervention (p-value 0.098).

Conclusions

The interprofessional education seminar and pocket reference sheet realigned clinical practice with guideline-based therapy in this tertiary care, community hospital. These data validate that learner-centric innovation will benefit patient outcomes and improve the educational potential of the interdisciplinary rounding team.

## Introduction

Chronic obstructive pulmonary disease (COPD) is a leading cause of chronic morbidity and mortality worldwide and currently ranks as the fourth leading cause of death worldwide - third in the United States [1-2]. Projections estimate that COPD will rise to the third most common cause of death worldwide by the year 2020 [1,3]. COPD affects nearly 24 million Americans nationwide; 70% of those 24 million are under the age of 65 and many more are undiagnosed [4]. As medical knowledge advances and the average life span lengthens, the prevalence of COPD will rise, entailing a larger treatment demographic requiring increasing medical therapy and health care costs. This increase poses a large burden on society and the healthcare system. COPD is a major growing cause of morbidity and mortality worldwide. The cost of care, both direct and indirect, related to COPD resulted in $49.9 billion in 2010; by 2020, COPD is projected to rank fifth in the burden of disease worldwide [2]. Estimates suggest approximately 40% of all COPD costs could be avoided by preventing complications and hospitalizations [4]. Research is currently focused on novel treatments and preventative therapies to help manage COPD and mitigate exacerbations.

The pathogenesis and clinical presentation of COPD are multifactorial, and ongoing research aims to identify risk factors and other mechanisms of exacerbating factors to better treat and manage the illness [2,5]. Patients with COPD average four, concurrent, co-morbid medical conditions and take five to 10 different medications [6]. A diverse range of severity within the disease compounds an already heterogeneous population. The Global Initiative for Chronic Obstructive Lung Disease (GOLD) has stratified the disease into classes and grades. There are four subtypes of severity based on physiologic, functional data, and further subdivision based on symptom severity and frequency [2]. Cohorted patients residing in each COPD class require specific treatment regimens to achieve the desired therapeutic outcome [7]. Additionally, these treatment regimens are further modified to reach treatment goals based on the present co-morbid conditions. At present, therapies such as smoking cessation and supplemental oxygen are the only clear strategies for reducing disease progression and mortality [5].

Currently, this tertiary-care, community hospital aims to employ guideline-directed medical therapy for all of our patients. The institution has not performed a comprehensive review analyzing compliance with the GOLD guideline-based systemic corticosteroid treatment during acute exacerbations of COPD. Defining an exacerbation as an acute clinical and symptomatic worsening of a patient’s respiratory symptoms resulting in medication change and medical intervention, this retrospective review will analyze the current hospital practice of systemic steroid administration during COPD exacerbation treatment [1]. These data will allow our hospital system clinicians to garner a greater understanding of treatment objectives, side-effects, and outcomes related directly to acute COPD exacerbation treatment choices. Clinical Learning Environment Review (CLER) studies further support the conclusion that local, community patients require treatment optimization. Therefore, these studies will promote utilizing guideline-directed therapy in the care of Cape Fear Valley Medical Center patrons in Fayetteville, North Carolina [8].

A COPD exacerbation diagnosis relies clinically on both patient presentation and provider intuition. Acute changes, beyond normal respiratory variation, must be present: increased dyspnea, cough, and/or sputum production [1]. Approximately, 25% of these exacerbations require pharmacologic intervention [1,9-10]. Systemic corticosteroid therapy has been proven beneficial in multiple, randomized, multicenter trials. Corticosteroid administration is associated with accelerated recovery time, improvements in forced expiratory volume (FEV_1_) and arterial hypoxemia, and reduced hospital length of stay [11]. This therapy is routinely used and firmly supported within the GOLD guidelines. More recent trials evaluated the proper dosage and duration of corticosteroid administration. In 2013, the REDUCE trial demonstrated that five days of glucocorticoid therapy was non-inferior to 14 days on re-exacerbations within six months [12]. Following this trial, the recommendation of 40 milligrams (mg) of prednisone per day for a five-day duration was made. This was reflected in the 2014 GOLD guidelines and have continued to propagate through to the current guideline recommendations for the treatment of acute COPD exacerbations.

Despite these guideline recommendations, health care facilities frequently employ higher doses of systemic corticosteroid therapy for longer durations than currently recommended. Our study evaluated how the practitioners at Cape Fear Valley Medical Center, in Fayetteville, North Carolina, treated acute COPD exacerbations through a medication use evaluation (MUE). The investigative team then instituted an interprofessional, quality improvement initiative to monitor changes in practice and adherence to the GOLD guideline recommendations for the treatment of acute COPD exacerbations.

## Materials and methods

Trial design and oversight

This study was a two-phase, retrospective analysis of systemic corticosteroid therapy in acute COPD exacerbation at Cape Fear Valley Medical Center, an urban, tertiary care center, in Fayetteville, North Carolina. Analysis before and after the implementation of an interprofessional, learner-centric, educational intervention was obtained (Appendix B). Data were collected for the control and educational groups on Internal Medicine resident patients with a diagnosis of COPD exacerbation between October 2017 and February 2018. Metrics utilized to assess guideline compliance and steroid-related adverse events included mean corticosteroid daily dose, starting corticosteroid regimen dose, and elevations in blood pressure, blood glucose, and white blood cell count. The education initiative was given to all providers and pharmacists covering the resident, inpatient medical services. Comparisons were made to assess the efficacy of the proposed intervention on guideline compliance.

This study was approved for exemption by Cape Fear Valley Medical Center’s Institutional Review Board, conducted in compliance with the institutional review board-approved protocol, and all investigations were conducted in compliance with Good Clinical Practice and applicable regulatory guidelines.

Study population

Patients were included in the study if they were 18 years of age or older with a diagnosis of admission or observation for COPD exacerbation, as identified by International Classification of Diseases 10th Revision (ICD-10) code, 491.21 or 491.22, or Diagnosis Related Group (DRG) code J44.1, and received at least 24 hours of scheduled systemic corticosteroids. Charts with admission dates between October 2017 and February 2018 were reviewed as the control group. Charts with admission dates between October 2018 and February 2019 were included in the intervention education group following the September 2018 education initiative.

Patients were excluded from the study if any indication requiring systemic corticosteroid other than COPD exacerbation was found. Exclusionary conditions included, but were not limited to, chronic obstructive asthma with acute exacerbation, asthma with acute exacerbation, acute exacerbation of asthma with allergic rhinitis, rheumatoid arthritis, Addison’s disease, systemic lupus erythematosus, ulcerative colitis, Crohn’s disease, and sarcoidosis.

The first 300 charts to meet the inclusion criteria were reviewed for age, gender, systemic corticosteroid use, starting corticosteroid dose, total corticosteroid dose received, total prednisone dose equivalent, duration of corticosteroid therapy, hospital length of stay, and 30-day readmission rate.

Study endpoints: data collected

The primary endpoint of the study compared provider compliance with GOLD Guideline-directed corticosteroid usage in COPD exacerbations before and after the implementation of an interprofessionally designed, clinical education program. Results were divided into three groups: patients who received corticosteroids in compliance with the 2018 GOLD guidelines, those who received lower doses than recommended, and those who received higher doses than recommended.

The secondary endpoints of the study included the comparison of adverse events, length of stay, and readmission rates. Methods of prescribing were also described. Steroid-related adverse events included increases in blood glucose, blood pressure, and white blood cell count. Increases in blood glucose were defined as new or worsening fasting plasma glucose (FPG) > 100 mg/dL or random glucose > 140 mg/dL, increase in insulin requirements by 20% or greater, increase in the required dosage of an oral antihyperglycemic agent, or the addition of a new oral antihyperglycemic agent for blood glucose control. Increases in blood pressure (BP) were defined as new or worsening BP >140 mmHg systolic blood pressure (SBP), >90 mmHg diastolic blood pressure (DBP) or both, and the addition of one or more antihypertensive agents to the previous regimen. Lastly, increases in white blood cell count (WBC) were defined as an increase in WBC >20% between consecutive measurements after the initiation of glucocorticoid therapy.

Statistical analysis

Based on a two-sided alpha value of 0.5 with a 95% confidence interval, it was estimated that 129 patients per group would be required to reach 80% power, assuming an increase in GOLD guideline compliance of 20%. The primary endpoint of compliance with GOLD-guideline recommendations was analyzed using Pearson’s Chi-Squared analysis. Pearson’s Chi-squared was also used for nominal secondary endpoints, including adverse effects and readmissions, and the presence of baseline disease states. Student t-tests were used to compare hospital length of stay, mean corticosteroid dose, and patient age.

All statistical calculations were computed utilizing JMP-14 PRO (SAS, Cary, NC).

## Results

Trial population

The control group included 137 patients, 61% female, with a mean age of 65 years. The education group consisted of 105 patients meeting inclusion criteria, 60% female. The mean age for the education group patients was 67 years. There were no significant differences in key pre-existing conditions including diabetes and hypertension (Table 1).

**Table 1:** Patient baseline characteristics

Characteristic	Control Group (N = 137)	Education Group N = 105
Age, Years (mean + SD)	65.1 + 10.5	68.9 + 11.6
Gender, Female (n%)	84 (61.3)	63 (60)
Diabetes, n(%)	80 (58.4)	68 (64.8)
Hypertension, n(%)	98 (71.5)	81 (77.1)

Primary endpoint

The medication use review revealed that 90% of patients admitted or observed with the diagnosis of acute COPD exacerbation received methylprednisolone as the initial corticosteroid of choice with an initial median dose of 75 mg. The average daily dose was equivalent to 147 mg of prednisone. The analysis revealed 95% of patients reviewed received corticosteroid doses above the guideline-recommended dose of 40 mg of prednisone equivalents daily. Only 3% of the patients reviewed received guideline-directed therapy (Table 2).

**Table 2:** Primary and secondary endpoint analysis GOLD: Global Initiative for Chronic Obstructive Lung Disease

	Control Group N=137	Education Group N=105	P-Value
Primary Endpoint			
Mean Dose per Day	147.5 mg	58.0 mg	< 0.0001
GOLD guideline therapy started, n(%)	4 (2.9)	47 (44.8)	< 0.0001
Regimens > prednisone 40mg, n(%)	130 (94.9)	54 (51.4)	< 0.0001
Regimens < prednisone 40mg, n(%)	3 (2.2)	4 (3.81)	0.89
Secondary Endpoints			
Increased Blood Glucose, n(%)	85 (62.0)	33 (31.4)	< 0.0001
Increased Blood Pressure, n(%)	72 (52.6)	26 (24.8)	< 0.0001
Increased White Blood Cell Count, n(%)	74 (54.0)	35 (33.3)	0.014
Length of Stay, days (mean + SD)	6.1 + 4.1	4.7 + 2.8	0.009
30-Day Readmissions, n(%)	43 (31.4)	22/105 (21.0)	0.098

Following the interprofessional, quality improvement intervention of provider education and COPD pocket handouts, a statistically significant change in the administration of corticosteroid medications in the setting of acute COPD exacerbations was observed. The initial median starting dose, in prednisone equivalents, decreased from 75 mg daily to 50 mg following intervention (p-value <0.0001). There was also a reduction in the mean dose per day in prednisone equivalents from 147.5 mg per day to 58 mg per day (p-value < 0.0001). While 130 of 137 (94.9%) patients received corticosteroid doses above the GOLD guideline recommendations before the educational intervention, only 54 of 105 (51.4%) patients received doses above the GOLD recommendations in the post-intervention evaluation (p-value <0.0001). As the number of guideline-directed doses increased following the educational intervention, the number of patients with acute exacerbations of COPD receiving the recommended dose of 40 mg of prednisone daily increased from four of 137 (2.9%) to 47 of 105 (44.8%), achieving statistical significance at a p-value <0.001.

**Figure 1:**
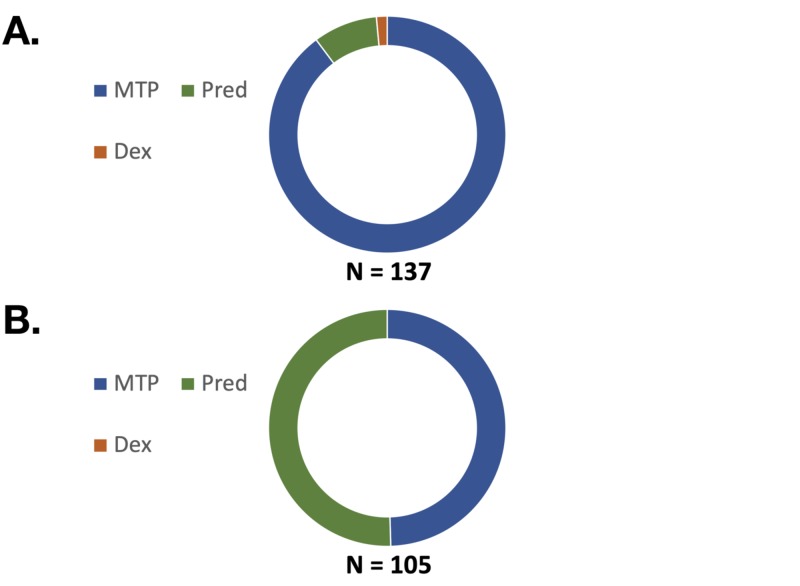
Initial steroid prescribed on admission pre and post-intervention MTP: Methylprednisolone; Pred: Prednisone; Dex: Dexamethasone A: 90% of patients initiated on Methylprednisolone (MTP); 9% of patients initiated on Prednisone (Pred); 1% of patients initiated on Dexamethasone (Dex) B: 49.5% of patients initiated on Methylprednisolone (MTP); 50.5% of patients initiated on Prednisone (Pred); 0% of patients initiated on Dexamethasone (Dex)

Overall, the use of prednisone as the initial drug therapy of choice increased significantly following the intervention (Figure 1). In the pre-intervention analysis, the majority of providers used methylprednisolone (90%) as their therapeutic choice for the management of acute COPD exacerbations. These figures were seen to normalize in the post-education analysis with 49.5% of patients receiving methylprednisolone and 50.5% receiving prednisone as the initial, scheduled systemic corticosteroid.

Secondary endpoints

The patients in both arms of the study were subjected to potential corticosteroid-related adverse drug reactions (ADRs): increased blood glucose measurements, increased blood pressure, and increased white blood cell count. Decreased corticosteroid dose administration, following the GOLD guideline implementation, provided statistically significant reductions in these ADRs. The percentage of patients with increased blood glucose fell from 85 of 137 (62.0%) to 33 of 105 (31.4%) following interprofessional education (p-value < 0.0001). Similar changes were seen in the percentage of patients who experienced increased blood pressure for corticosteroid administration. The percentage of patients with corticosteroid-related hypertension in the control group was 52.6% (72 of 137); this percentage fell to 24.8% (26 of 105), which was found to have statistical significance to a p-value < 0.0001. Leukocytosis, another well-known ADR of corticosteroid administration, was decreased by 20.6% (54.0% to 33.3%) from 74 patients in the control group to only 35 patients in the educational group (p-value 0.014).

Other indices examined during this study were hospital length of stay and 30-day hospital readmission rate. Hospital length of stay was decreased by 1.4 days with a reduced total daily dose of corticosteroids following the intervention (p-value 0.009). The only index not seen to meet statistical significance was the effect of GOLD guideline-directed corticosteroid use on a 30-day hospital readmission rate. In the control group, 43 of 137 patients studied (31.4%) were readmitted within 30 days of hospital discharge. Interprofessional education decreased this number to 22 of 105 (21.0%), which fell short of the 95% confidence interval (p-value 0.098).

## Discussion

This study found that among patients with an admission diagnosis of acute exacerbation of chronic obstructive pulmonary disease, improving adherence to GOLD guideline-directed use of systemic corticosteroid therapy was associated with a decrease in hyperglycemia, hypertension, and leukocytosis related to corticosteroid administration. These patients also demonstrated a decrease in overall hospital length of stay. These data indicate that resident-led education positively influenced guideline-directed medical therapy implementation for the management of acute COPD exacerbations.

Therapeutic recommendations during acute exacerbations of COPD were the focal point of evaluation during this study, specifically the recommendation for systemic corticosteroid usage for the resolution of acute COPD exacerbation. Multiple trials have investigated various doses, routes of administration, duration of therapy, and adverse effects of corticosteroid administration. Presently, the 2017 GOLD guidelines recommend an oral, five-day course of 40 mg prednisone [1]. The REDUCE trial published in 2013 proved that short-term treatment was non-inferior to conventional treatment concerning re-exacerbations within a six-month follow-up timeframe [12]. The REDUCE trial also showed a significant reduction in glucocorticoid exposure in the short-term treatment group and decreased treatment-associated adverse reactions were appreciated with the reduced dosage [12]. Further trials have shown that oral systemic corticosteroid administration has equivalent efficacy when compared to parenteral or intravenous (IV) administration [13]. There is also literature suggesting that corticosteroid doses between 30 mg and 100 mg significantly increase receptor saturation in a dose-dependent fashion: at doses approaching 100 mg of prednisone equivalents daily, 100% of genomic glucocorticoid effects are assumed to be exerted [14]. The quality improvement initiative utilized these facts regarding the use of systemic corticosteroid therapy in the management of acute exacerbations of COPD.

In the world of academia and medical education, interprofessional teams work together and strive to deliver the best, most up-to-date care possible for their patients. This learner-centric, interprofessional, quality improvement study resulted in a significant shift in clinical practice toward guideline, evidence-based medicine. This study helps validate the benefit of interdisciplinary rounding teams and speaks to the impact potential of an interprofessional team on patient care. Engaging learners to invest in their education as it pertains to patient care is an ever-evolving model of medical education. Learner-centric education initiatives stand at the heart of this medical education modality.

These data reflect the growing body of evidence that low-dose, systemic corticosteroid therapy is non-inferior to an alternative regimen of the higher dose, parenteral corticosteroid administration. These data also reflect the abundant success of interprofessional education in clinical practice. Learner-driven patient care, as demonstrated by this study, provides meaningful health care to patients and education to learners. The interprofessional model provides multimodal care for maximal patient benefit.

Limitations

This study is not without limitations. As the study was conducted retrospectively, establishing causation of our secondary endpoints by corticosteroid administration could not be done. Additionally, dose-dependent connections between corticosteroid administration and ADRs were not established. These two limitations are perhaps the most notable as we concluded our secondary endpoint analysis. Also, our educational seminar was attended only by interprofessional rounding teams consisting of internal medicine residents and pharmacy residents. Other hospitalists, although invited, were not required to attend the lecture nor did they receive the reference material at the onset of the study. As the improvement initiative was not implemented universally throughout the attending physician faculty, the generalizability of these results across the entirety of the hospital may be limited. Last, as this initiative was carried out primarily by residents, therapeutic decisions ultimately remained in the hands of each patient’s attending physician of record. This fact may have skewed some of the therapeutic data away from our primary objective of 40 mg of prednisone equivalents daily.

## Conclusions

The utilization of an interprofessional, educational, quality improvement initiative to increase adherence to guideline-directed medical therapy and the application of evidence-based medicine in a tertiary-care community hospital was successful in increasing GOLD guideline compliance and reducing corticosteroid-related adverse events. The interprofessionally developed education seminar and pocket reference sheet decreased the overuse of corticosteroids in our institution. These data speak to the effectiveness of interprofessional education and learner-centric care protocols. As this protocol was developed by learners for the education of their peers and other providers by proxy, not only did this study improve patient care, but it also improved the education of the learners, residents and students alike. Continuing collaborative care efforts such as this can have a wide-reaching impact on medical education and care of patients. The results were somewhat predictable based on previously defined studies and reviews in the literature, but the true benefit resides in the educational and academic capacity with which the learners implemented profound change in hospital practice. As the landscape of medicine continues to rapidly change, implementing cutting edge ideas via learner-driven initiatives will be paramount to the advancement of the medical community, academic and non-academic providers alike.

## Appendices

Appendix A: Abbreviations

ADR: Adverse Drug Reaction

BP: Blood Pressure

CAT: Chronic Obstructive Pulmonary Disease Assessment Test

CLER: Clinical Learning Environment

COPD: Chronic Obstructive Pulmonary Disease

DBP: Diastolic Blood Pressure

FEV_1_: Forced Expiratory Volume in 1 Second

FEV_1_/FVC: Ratio of Forced Expiratory Volume in 1 Second to Forced Vital Capacity

FVC: Forced Vital Capacity

FPG: Fasting Plasma Glucose

FRC: Functional Residual Capacity

GOLD: Global Initiative for Chronic Obstructive Lung Disease

HR: Hazard Ratio

ICS: Inhaled Corticosteroids

IV: Intravenous

LABA: Long-Acting Beta-2 Agonist

LAMA: Long-Acting Antimuscarinic Antagonist

MD: Mean Absolute Difference

mg: Milligram

mMRC: Modified British Medical Research Council

MUE: Medication Use Evaluation

NHLBI: National Heart, Lung, and Blood Institute

NNT: Number Needed to Treat

OR: Odds Ratio

PDE-4: Phosphodiesterase-4

PO: Per Os (by mouth/oral)

PRN: Pro Re Nata (as needed)

RV: Residual Volume

SABA: Short-Acting Beta-2 Agonist

SBP: Systolic Blood Pressure

TLC: Total Lung Capacity

WBC: White Blood Cell Count

WHO: World Health Organization

Appendix B: Interprofessional education initiative handout

Management of an Acute Exacerbation of Chronic Obstructive Pulmonary Disease

Key points:^1^

- An exacerbation of COPD is defined as an acute worsening of respiratory symptoms necessitating additional therapy.

- Exacerbations are multifactorial in originating cause.

- The most common causes are respiratory tract infections.

- Minimizing the current deleterious functional impact of the exacerbation and prevention of future exacerbations are the goals of treatment.

- Initial respiratory inhaler therapy should begin with short-acting inhaled beta-2 agonists (SABA), with or without short-acting anticholinergics

- Maintenance therapy with long-acting bronchodilators (LABA) or long-acting antimuscarinic (LAMA) medication should be initiated as soon as possible before hospital discharge. Maintenance therapy should be tailored to the patient's specific disease burden and needs.

- Systemic corticosteroid administration is recommended for improvement in lung function (FEV_1_), improvement in oxygenation and reduction in both recovery time and duration of hospitalization. Duration should not exceed five to seven days.

- Antibiotics, when indicated, may shorten recovery time, reduce the risk of early relapse, treatment failure, and hospitalization duration. As with steroids, the duration of therapy should be five to seven days.

- Methylxanthines are no longer recommended due to their side-effect profiles.

- Non-invasive mechanical ventilation should be the first mode of ventilation used in COPD patients with acute respiratory failure who have no absolute contraindication because it improves gas exchange, reduces the work of breathing and the need for intubation, decreases hospitalization duration and improves survival.

Systemic Corticosteroids versus placebo:^2^

- Nine studies with 921 participants median treatment duration = 14 days

Corticosteroids reduce treatment failure

  OR 0.48 (95% CI, 0.35 to 0.67)

NNT = Nine people treated (95% CI, 7 to 14 people) to avoid one treatment failure

- Two studies with 415 participants

  Lower rate of relapse at one month

  HR 0.78 (95% CI, 0.63 to 0.97)

- Twelve studies with 1319 participants

Corticosteroids DO NOT impact mortality

  OR 1.00 (95% CI, 0.60 to 1.66)

- Seven studies with 649 participants

Corticosteroids improve FEV_1_

  140 mL (95% CI, 90 to 200 mL)

Corticosteroids were associated with increased adverse reactions

  OR 2.33 (95% CI, 1.59 to 3.43)

  NNH = 6 people treated (95% CI, 4 to 10) will result in one extra adverse event

  Hyperglycemia- OR 2.79 (95% CI, 1.86 to 4.19)

Decrease general inpatient hospitalization duration

  Duration -1.22 days (95% CI, -2.26 to -0.18)

  No difference in intensive care unit hospitalization

Oral versus parenteral:

- No significant difference in the primary outcomes of treatment failure, relapse, or mortality or for any secondary outcomes^3^

  Increases in adverse effects were greater with parenteral compared with oral treatment.

  Increased hyperglycemia with parenteral in one study

  OR 4.89 (95% CI, 1.20 to 19.94)

- One study with 79985 participants^4^

The risk of treatment failure, length of stay, and the cost were significantly lower among orally treated patients when compared to parenteral treatment in a propensity-matched analysis.

- One study with 435 participants:^5^

 Therapy with oral prednisolone is not inferior to IV treatment in the first 90 days after starting therapy.

  Overall treatment failure within 90 days was similar

  No differences in early (within two weeks) treatment failure or late (after two weeks) treatment failure

  No difference in the mean length of hospital stay

Over one week, clinically relevant improvements were found in spirometry and health-related quality of life, without significant differences between oral and parenteral treatment groups.

Duration of therapy:

- Eight studies with 582 participants^6^

 Four studies revealed no difference in risk of treatment failure between short and long duration systemic corticosteroid treatment

  OR 0.72 (95%, CI 0.36 to 1.46)

  NNT = 22 fewer per 1000 for short-duration treatment (95% CI, 51 fewer to 34 more)

No difference in risk of relapse

  OR 1.04 (95% CI, 0.70 to 1.56)

  NNT = Nine fewer per 1000 for short-duration treatment (95% CI 68 fewer to 100 more)

- One study with 314 participants^7^

Non-inferiority between five and 14 days of corticosteroid treatment with respect to time to next exacerbation

  HR 0.95 (95% CI, 0.66 to 1.37)

- Five studies with 503 participants

No difference in the likelihood of adverse events between short and longer duration corticosteroid therapy

  OR 0.89, 95% CI 0.46 to 1.69

  NNT = nine fewer per 1000 (95% CI 44 fewer to 51 more)

- Length of hospital stay (evaluated in 421 participants)

No difference between short and longer duration corticosteroid treatment

  MD -0.61 days (95% CI, -1.51 to 0.28 days)

- Lung function (evaluated in 185 participants)

No difference between short and longer duration corticosteroid treatment

  MD FEV_1_ -0.04 L (95% CI, -0.19 to 0.10)

- One study with 271 participants^8^

Two-week versus 8-week corticosteroid therapy

  Maximal benefit is obtained during the first two weeks of therapy

  Hyperglycemia requiring treatment is the most frequent complication

  Significant treatment benefits were no longer evident at six months

Summary

- The current recommendation for the treatment of acute exacerbation of chronic obstructive pulmonary disease is a short-course, low-dose oral corticosteroids: Prednisone 40 mg by mouth once daily for five days

- No taper is needed for this regimen

Footnotes

^1^ Vogelmeier CF, Criner GJ, Martinez FJ, et al. Global Strategy for the Diagnosis, Management, and Prevention of Chronic Obstructive Lung Disease 2017 Report. GOLD Executive Summary. Am J Respir Crit Care Med. 2017;195(5):557-582.

^2^ Walters JAE, Tan DJ, White CJ, Gibson PG, Wood-Baker R, Walters EH. Systemic corticosteroids for acute exacerbations of chronic obstructive pulmonary disease. Cochrane Database of Systematic Reviews 2014, Issue 9. Art. No.: CD001288. DOI: 10.1002/14651858.CD001288.pub4.

^3^ Hernandez JM, Edmonds M. Do systemic corticosteroids improve outcomes in chronic obstructive pulmonary disease exacerbations? Annals of Emergency Medicine 2016;67(2):258-259.

^4^ Lindenauer PK, Pekow PS, Lahti MC, Lee Y, Benjamin EM, Rothberg MB. Association of corticosteroid dose and route of administration with risk of treatment failure in acute exacerbation of chronic obstructive pulmonary disease. JAMA. 2010;303(23):2359-67.

^5^ De jong YP, Uil SM, Grotjohan HP, Postma DS, Kerstjens HA, Van den Berg JW. Oral or IV prednisolone in the treatment of COPD exacerbations: a randomized, controlled, double-blind study. Chest. 2007;132(6):1741-7.

^6^ Walters JAE, Tan DJ, White CJ, Wood-Baker R. Different durations of corticosteroid therapy for exacerbations of chronic obstructive pulmonary disease. Cochrane Database of Systematic Reviews 2018, Issue 3. Art. No.: CD006897. DOI: 10.1002/14651858.CD006897.pub4.

^7^ Leuppi JD, Schuetz P, Bingisser R, et al. Short-term vs conventional glucocorticoid therapy in acute exacerbations of chronic obstructive pulmonary disease: the REDUCE randomized clinical trial. JAMA. 2013;309(21):2223-31.

^8^ Niewoehner DE, Erbland ML, Dupree RH, et al. Effect of systemic glucocorticoids on exacerbations of chronic obstructive pulmonary disease. N Engl J Med 1999;340:1941-7.
